# Angiogenic and Fibrogenic Dual-effect of Gremlin1 on Proliferative Diabetic Retinopathy

**DOI:** 10.7150/ijbs.85735

**Published:** 2024-01-12

**Authors:** Xinjing Wu, Bing Qin, Ruiwen Cheng, Ru Zhou, Xingxing Wang, Zhengyu Zhang, Xiying Mao, Zhan Xie, Mingkang Chen, Lin Jiang, Ping Xie, Jiangdong Ji, Weiwei Zhang, Songtao Yuan, Zizhong Hu, Qinghuai Liu

**Affiliations:** 1 Department of Ophthalmology, The First Affiliated Hospital of Nanjing Medical University, Nanjing 210029, China.; 2 Department of Ophthalmology, The Affiliated Suqian First People's Hospital of Nanjing Medical University, Suqian 223800, China.; 3 Department of Ophthalmology, People's Hospital of Yangzhong City, Yangzhong 212200, China.; 4 Department of Endocrinology, The First Affiliated Hospital of Nanjing Medical University, Nanjing 210029, China.

**Keywords:** Gremlin1, Angiogenesis, Microglia, Fibrosis, Proliferative diabetic retinopathy

## Abstract

Ocular angiogenic diseases, such as proliferative diabetic retinopathy (PDR), are often characterized by pathological new vessels and fibrosis formation. Anti-vascular endothelial growth factor (VEGF) therapy, despite of its efficiency to inhibit new vessels, has limitations, including drug resistance and retinal fibrosis. Here, we identified that Gremlin1, a novel angiogenesis and fibrosis inducer, was secreted from Müller glial cells, and its expression increased in the vitreous fluid from patients with PDR. Mechanistically, Gremlin1 triggered angiogenesis by promoting endothelial-mesenchymal transition via the EGFR/RhoA/ROCK pathway. In addition, Gremlin1 activated microglia to present profibrotic and fibrogenic properties. Further, anti-Gremlin1 antibody inhibited ocular angiogenesis and microglia fibrosis in mouse models. Collectively, Gremlin1 could be a potential therapeutic target in the treatment of ocular angiogenic diseases.

## Introduction

In the microenvironment of diabetic retinae, chronic hyperglycemia gradually causes damage to the retinal vasculature, finally leading to neovascularization 1. In addition to vascular lesions, the concept that neurodegenerative changes are one of the early events of DR has become a consensus 2, 3. Proliferative diabetic retinopathy (PDR), as the end stage of DR, is characterized by retinal neovascularization and fibrotic change at the vitreoretinal interface. If untreated, subsequent vitreous hemorrhage, retinal fibrosis, tractional retinal detachment, and neovascular glaucoma may occur and lead to blindness 4. Vascular endothelial growth factor (VEGF) plays an important role in angiogenesis. During the past decade, intravitreal injection of anti-VEGF agents has been widely used to manage DR, but its efficacy is limited by various causes, such as resistance to anti-VEGF therapy, retinal atrophic and fibrotic changes after multiple injections, and difficulties for patients and physicians with frequent often monthly injections, and significant economic costs for patients and the healthcare system 5. These challenges call for novel and multi-target interventional strategies.

Gremlin1 is a 23 to 28 kDa glycosylated protein from the family of bone morphogenetic protein (BMP) antagonists. Typically, Gremlin1 interacts with BMP to regulate the development of bones and kidneys 6. Of note, Gremlin1 has been reported to be involved in both angiogenic and fibrogenic conditions, such as cancer 7, pulmonary arterial hypertension 8, and liver and renal fibrosis 9, yet with limited stress in ocular angiogenesis. We previously demonstrated that Gremlin1 induces epithelial-mesenchymal transition (EMT) 10 of retinal pigment epithelial cells. Considering its pro-EMT characteristics, we proposed the hypothesis that Gremlin1 induces pathological ocular angiogenesis by promoting endothelial-mesenchymal transition (EndMT) of endothelial cells (ECs), similar to its pro-angiogenic effect in human pulmonary artery endothelial cells 8.

Recently, studies have also suggested that microglia/macrophage participates in the formation of pathological fibrosis 11. We previously revealed that in the fibrovascular membranes (FVMs) of PDR, microglia, which presents both profibrotic and fibrogenic properties, is the main cell population. Also, it would be intriguing to investigate if Gremlin1 has a role in microglia-related retinal fibrosis.

In the current study, we focused on the Gremlin1 expression, function, and mechanism during pathological retinal angiogenesis and fibrosis. We further evaluated the therapeutic potential of anti-Gremlin1 antibody in the treatment of pathological angiogenesis as well.

## Results

### Gremlin1 expression elevated in ocular angiogenesis mouse models and in vitreous fluid of PDR patients

**Figure 1:**
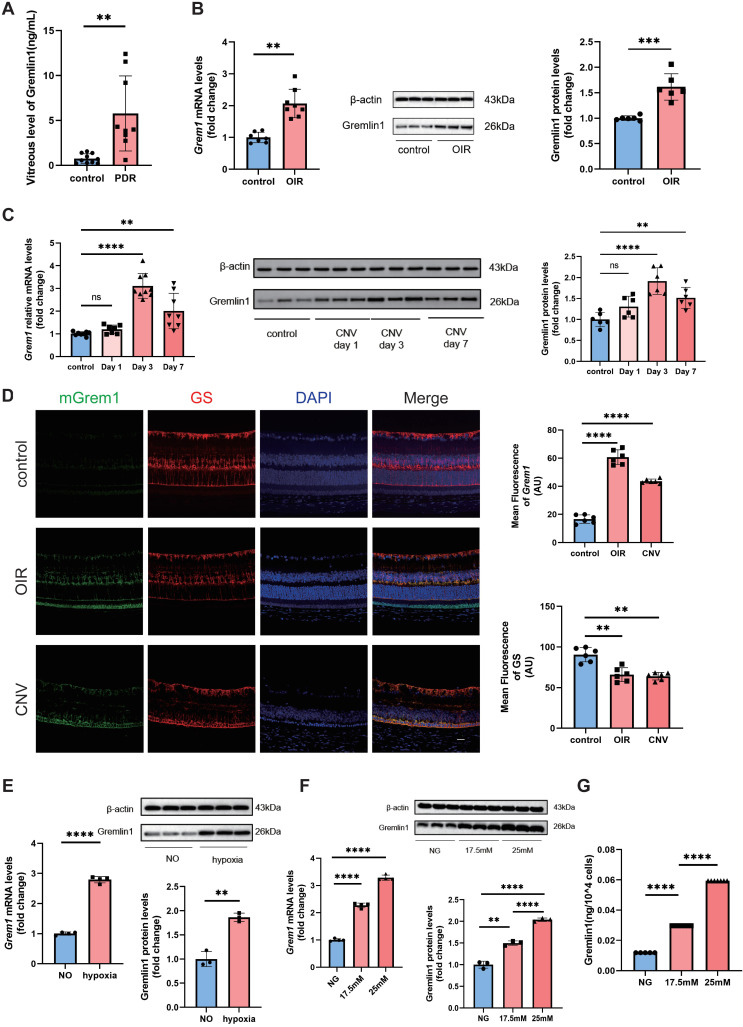
** Elevated Gremlin1 expression in vitreous fluid of patients with PDR and in mouse models. (A)** Quantification of Gremlin1 protein levels by multiplex liquid-chip assay analysis (n=10 for control subjects, n=9 for PDR patients). **(B)** Quantification of *Grem1* mRNA (n=7-8, *Gapdh* was used as the internal control) and Gremlin1 protein expression levels (n=6, β-actin was used as the internal control) in the retinae of OIR and control groups. Student's* t* test was used to compare differences. **(C)** Quantification of *Grem1* mRNA (n=7-9, *Gapdh* was used as the internal control) and Gremlin1 protein expression levels (n=6, β-actin was used as the internal control) in CNV mice model. Student's* t* test was used to compare differences. One-way ANOVA was used to compare the difference. **(D)** Representative images of fluorescence *in situ* hybridization of *Grem1*(green) in the retinae with GS (red) and DAPI (blue). All three channels were merged. Scale bar, 50 μm. **(E)** Analysis of *Grem1* mRNA (n=4, *Gapdh* was used as the internal control) and Gremlin1 protein levels (n=3, β-actin was used as the internal control) in Müller cells treated as indicated. NO, normal oxygen. Student's* t* test was used to compare differences. **(F)** RT-PCR analysis assessed the *Grem1* mRNA levels (n=3-4,* Gapdh* was used as the internal control). Gremlin1 protein levels were quantified by Western blotting analysis (n=3, β-actin was used as the internal control). NG, normal glucose (5.5 mM). One-way ANOVA was used to compare the difference. **(G)** Level of soluble Gremlin1 in the Müller cells culture medium after high glucose treatment (n=5). NG, normal glucose (5.5 mM). One-way ANOVA was used to compare the difference. *P < 0.05, **P<0.01, ****P<0.0001 compared with control group.

To identify whether Gremlin1 contributes to retinal angiogenesis, we first evaluated its expression in vitreous fluid from 9 patients with PDR and 10 control patients (epiretinal membrane [ERM] or macular hole). The ELISA assay revealed that the concentration of Gremlin1 was significantly higher in vitreous humor of PDR patients than that of control patients (Figure 1A).

We also evaluated the expression of Gremlin1 in oxygen-induced retinopathy (OIR) and laser-induced choroidal neovascularization (CNV) mouse models. The retinal mRNA and protein levels of Gremlin1 was significantly elevated in OIR model, compared to that in aged-matched room air control pups (Figure 1B). Gremlin1 expression level was also reproducibly increased in the retinae of mice at 3 or 7 days after laser exposure, compared to that in controls (Figure 1C).

### Gremlin1 was secreted by Müller cells

To determine the source of Gremlin1, we carried out fluorescence *in situ* hybridization (FISH) of eye-cup sections in both OIR and CNV mouse models. FISH analysis revealed that *Grem1* was mainly localized in glutamine synthetase (GS)-positive Müller cells (Figure 1D).

In* in vitro* studies, after hypoxia treatment for 48h, the expression level of Gremlin1 increased significantly in Müller cells (Figure 1E). We also detected the effect of high glucose stimulation on primary retina Müller cells. PCR analysis conformed that the mRNA level of *Grem1* elevated in Müller cells after high glucose treatment. Meanwhile, both Western blotting and ELISA confirmed that the protein level of Gremlin1 in Müller cell culture medium also increased after high glucose exposure (Figure 1F, G).

Recently, Federica et al. have reported that *Grem1* was upregulated starting from day 3 after laser exposure in mice retina Müller cells (Figure S1A) 12. In addition, the mRNA-seq also indicated that the mRNA level of *Grem1* increased evidently in primary rat Müller cells stimulated with high glucose (Figure S1B). Moreover, single-cell transcriptome analysis consistently showed that *Grem1* was mainly expressed in the retinal Müller cells of Akimba mice which displays retinal angiogenesis and fibrosis. (Figure S1C) 13. These data suggested that Müller cells were the main source of Gremlin1 under retinal neovascularization.

### Gremlin1 promoted angiogenesis *in vitro* and *in vivo*

**Figure 2:**
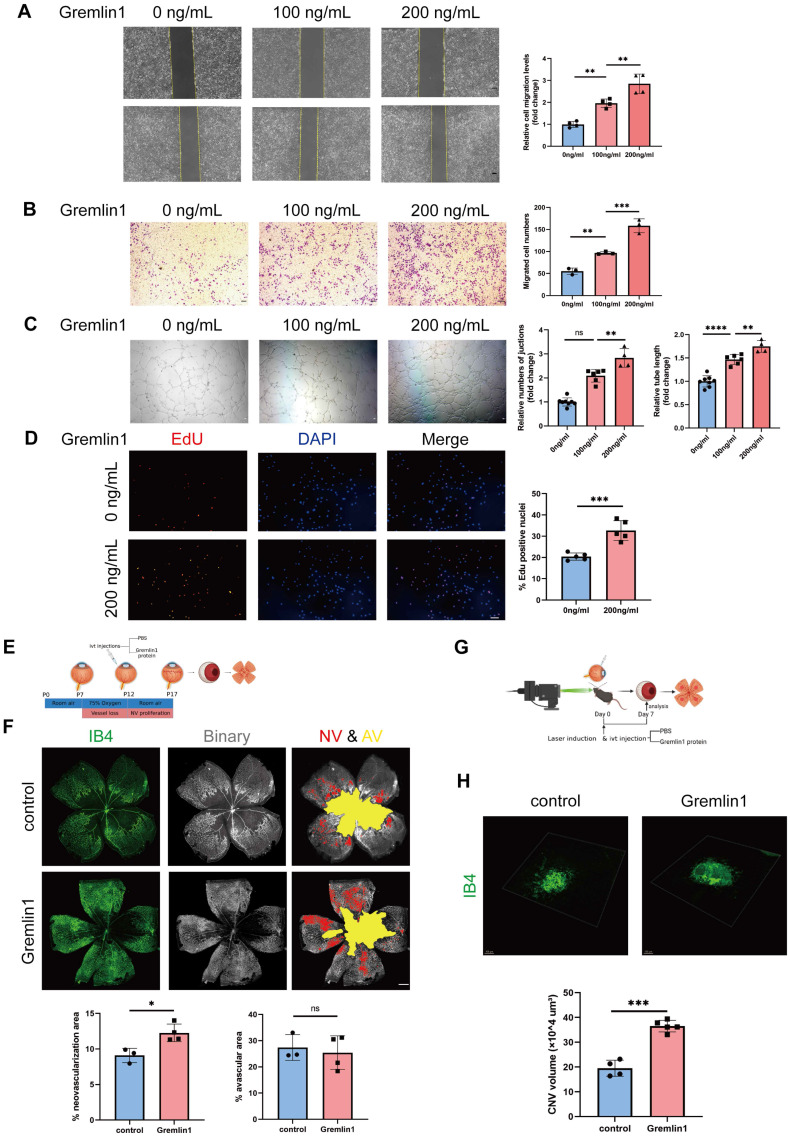
** Gremlin1 promoted angiogenesis both *in vivo* and *in vitro*. (A)** The migration of HURECs was detected by wounding healing assay at 0h and 24h after stimulation. The vertical yellow lines indicate the border of the wound. Average migration distance was calculated by gap width change (n = 4). One-way ANOVA was used to compare the difference. Scale bar, 50 μm. **(B)** Transwell invasion assay was performed to detect cell invasion. The number of migrated cells was analyzed (n = 3 for each group). One-way ANOVA was used to compare the difference. Scale bar, 50 μm. **(C)** HURECs were reseeded on the matrigel matrix and allowed to form tubes for 12h. The relative number of junctions and total length of tubes in each field were calculated (n=4-6). One-way ANOVA was used to compare the difference. Scale bar, 50 μm. **(D)** Representative images and quantification of EdU staining of HURECs cultured in different medium (n=5). Student's* t* test was used to compare differences. Scale bar, 50 μm. **(E, F)** Isolectin B4 staining was performed to examine pathological retinal neovascularization in whole-mount retinae at P17 in the OIR mice model. Percentages of neovascularization and avascular areas were calculated and compared (n=6). Student's* t* test was used to compare differences. Scale bar, 500 μm. **(G, H)** Isolectin B4 staining was performed to examine choroidal neovascularization in laser induced mouse CNV model. Choroidal tissue was scanned and reconstructed under a confocal microscope. Quantification of the volume of CNV (n=6 for each group and 4 laser burns per sample). Student's* t* test was used to compare differences. **P<0.01, ***P<0.001, ****P<0.0001 compared with control group.

Next, we investigated the effect of Gremlin1 on angiogenesis *in vitro*. Wound healing assay showed that the migration of human retinal endothelial cells (HURECs) was significantly enhanced after stimulation with Gremlin1 protein (200ng/mL) (Figure 2A). Transwell invasion assay showed that Gremlin1 significantly increased HURECs invasion (Figure 2B). Moreover, Gremlin1 strongly promoted tube formation in HURECs on Matrigel, as quantified by the number of junctions and tube length (Figure 2C). To determine the role of Gremlin1 in endothelial proliferation, HURECs were cultured in complete Endothelial Cell Medium (ECM) in the presence or absence of recombinant Gremlin1 protein for 24h, followed by a 2h EdU pulse. After the addition of Gremlin1, the number of EdU-positive HURECS increased significantly (Figure 2D).

We next examined the *in vivo* effect of pro-angiogensis of Gremlin1 on OIR model (Figure 2E). Intravitreal injections of Gremlin1 cytokines (200μg/mL) after induction of OIR led to a significant increase in neovascularization (Figure 2F). Moreover, we investigated the effect of Gremlin1 in CNV model (Figure 2G). The Isolectin B4 (IB4) staining of choroidal flat-mounts at 7 days after laser-induced CNV showed that Gremlin1 protein significantly increased the volume of CNV (Figure 2H).

### Gremlin1 induced EndMT in HURECs

**Figure 3:**
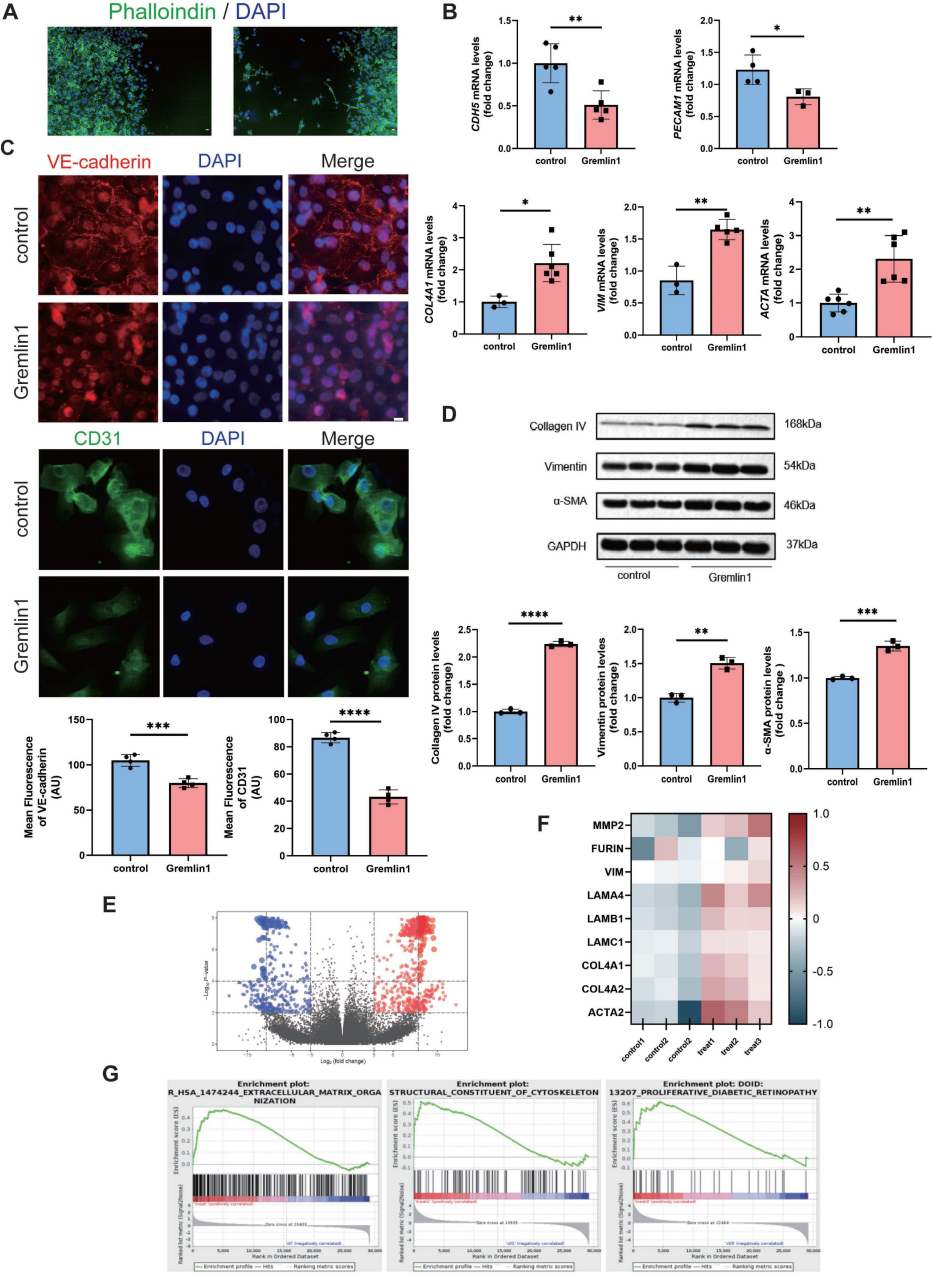
**Gremlin1 induced EndMT in HURECs. (A)** Phalloidine (green) staining showed the spindle shapes and lamellipodia of HURECS. Scale bar, 10 μm. **(B)** RT- PCR analysis assessed the mRNA level of endothelial markers *CDH5*,* PECAM*, *COL4A1*, *VIM* and *ACTA*. *GAPDH* was used as the internal control (n = 3-5 for each group). **(C)** Representative confocal images showed the expression of VE-cadherin (red), CD31(green) and DAPI (blue) in HURECs. Images of two channels were merged. Scale bar, 10 μm. **(D)** Representative western blots and quantification of Collagen type IV, Vimentin and α-SMA. GAPDH was used as the internal control (n = 3 for each group). **(E)** Volcano plot depicting the up (red) and down (blue)-regulated DEGs and non-DEGs (grey dots) according to log2 fold change and adj. p-value. **(F)** Heat map displayed the expression of typical genes involved in EndMT. **(G)** GSEA of the Reactome terms related to “Extracellular Matrix Organization”, “Structural Constituent of Cytoskeleton” and “Proliferative Diabetic Retinopathy”. Student's* t* test was used to compare differences. *P<0.05, **P<0.01, ***P<0.001 compared with control group.

We first treated HURECs with Gremlin1 protein (200ng/mL). After 24h, the HURECs demonstrated spindle shapes and increased lamellipodia (Figure 3A). Next, the expression levels of several EndMT-related markers were verified by RT-PCR and Western blotting analysis. As shown in Figure 3B, Gremlin1 significantly downregulated the expression of endothelial marker *CDH5* and *PECAM1*, and upregulated the expression of mesenchymal markers *COL4A1*, *VIM* and *ACTA*. Similar results were observed in immunofluorescence staining of VE-cadherin and CD31 (Figure 3C). Consistently, Western blotting analysis showed that the protein levels of Collagen type IV, Vimentin and α-SMA were significantly increased in HURECs after exposure of Gremlin1(Figure 3D).

Furthermore, whole transcriptome was measured using bulk RNA-seq after HURECs were exposed to 200 ng/mL Gremlin1 (Submission ID: SUB12858768; BioProject ID: PRJNA933390) (Figure 3E). Consistent with our previous results, changes were observed in several transcripts related to extracellular matrix organization (Figure 3F). Moreover, Gene Set Enrichment Analysis (GSEA) revealed enrichment of Gremlin1 in “Extracellular Matrix Organization”, “Structural Constituent of Cytoskeleton”, and “Proliferative Diabetic Retinopathy” (Figure 3G). Our data suggested that Gremlin1 acted HURECs through ECM degradation and remodeling, and cytoskeleton arrangement. This finding confirmed the up-regulated migration and invasion of HURECs, and implied the involvement of Gremlin1 in cytoskeletal rearrangement.

### Gremlin1 led to microglia-related fibrosis

**Figure 4:**
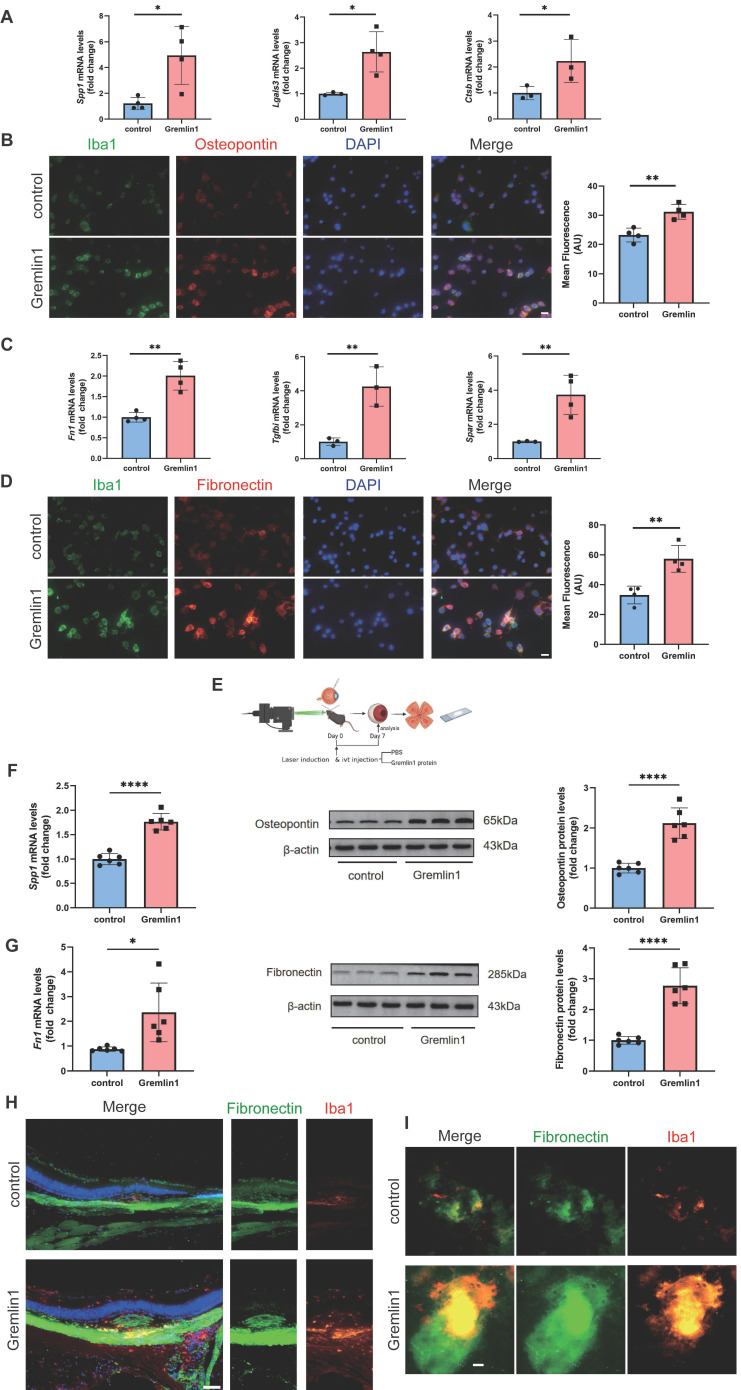
** Microglia stimulated by Gremlin1 represent both profibrotic and fibrogenic properties. (A)**RT-PCR analysis assessed the mRNA level of profibrotic properties genes. *Gapdh* was used as the internal control (n = 3-4 for each group). **(B)** Representative images and quantification of mean fluorescence of Osteopontin staining in BV2 cells (n=4). Three channels were merged. Scale bar, 10 μm. **(C)** RT-PCR analysis assessed the mRNA level of genes associated with extracellular matrix. *Gapdh* was used as the internal control (n = 3-4 for each group). **(D)** Representative images and quantification of mean fluorescence of Fibronectin staining in BV2 cells (n=4). Three channels were merged. Scale bar, 10 μm. **(E)** CNV modeling schematic. **(F)** Analysis of *Spp1* mRNA (n=6, *Gapdh* was used as the internal control) and Osteopontin protein levels (n=6, β-actin was used as the internal control) in mice retinas. **(G)** Analysis of *Fn1* mRNA (n=6, *Gapdh* was used as the internal control) and Fibronectin protein levels (n=6, β-actin was used as the internal control) in mice retinae. **(H)** Wax-embedded sections were stained for Fibronectin (green), Iba1 (red) and DAPI (blue). Three channels were merged. Scale bar, 100 μm. **(I)** RPE/choroid flatmounts were stained for Fibronectin (green) and Iba1 (red). Two channels were merged. Scale bar, 50 μm. Student's* t* test was used to compare differences. *P < 0.05, **P<0.01 compared with control group.

Microglia/macrophage has been reported to participate in the formation of pathological fibrosis 11. We next analyzed the effect of Gremlin1 on microglia-related fibrosis. RT-PCR showed that genes regulating the fibrosis activation, such as *Spp1*,* Lgals3* and *Ctsb*, were significantly upregulated after Gremlin1 treatment (Figure 4A) in BV2 cells. Correspondingly, microscopic investigation confirmed that Gremlin1 promoted the expression of Spp1-coded Osteopontin (Figure 4B). We also detected the role of Gremlin1 in activating microglia to be fibrogenic. Gremlin1-induced upregulation of* Fn1*, *Tgfbi* and *Spar* was observed by qPCR analysis (Figure 4C). The elevated expression of fibrogenic properties in Gremlin1-treated BV2 cells was further confirmed by immunofluorescence staining (Figure 4D). These data collectively imply a critical function of the Gremlin1 in the development of microglia-related fibrosis.

In laser-induced CNV in mice eyes (Figure 4E), the expression levels of *Spp1*, Osteopontin (Figure 4F),* Fn1* and Fibronectin (Figure 4G) were significantly increased. Immunofluorescence uncovered that the Fibronectin (FN)+ area and the number of FN+ microglia obviously elevated in the Gremlin1 protein-treated group (Figure 4H). Moreover, intravitreal injection of Gremlin1 increased Fibronectin+ area and obvious colocalization of Iba1 and Fibronectin was detected (Figure 4I).

### Activation of EGFR/MEK/Erk/RhoA/ROCK signaling in HURECs and microglia by Gremlin1 stimulation

**Figure 5:**
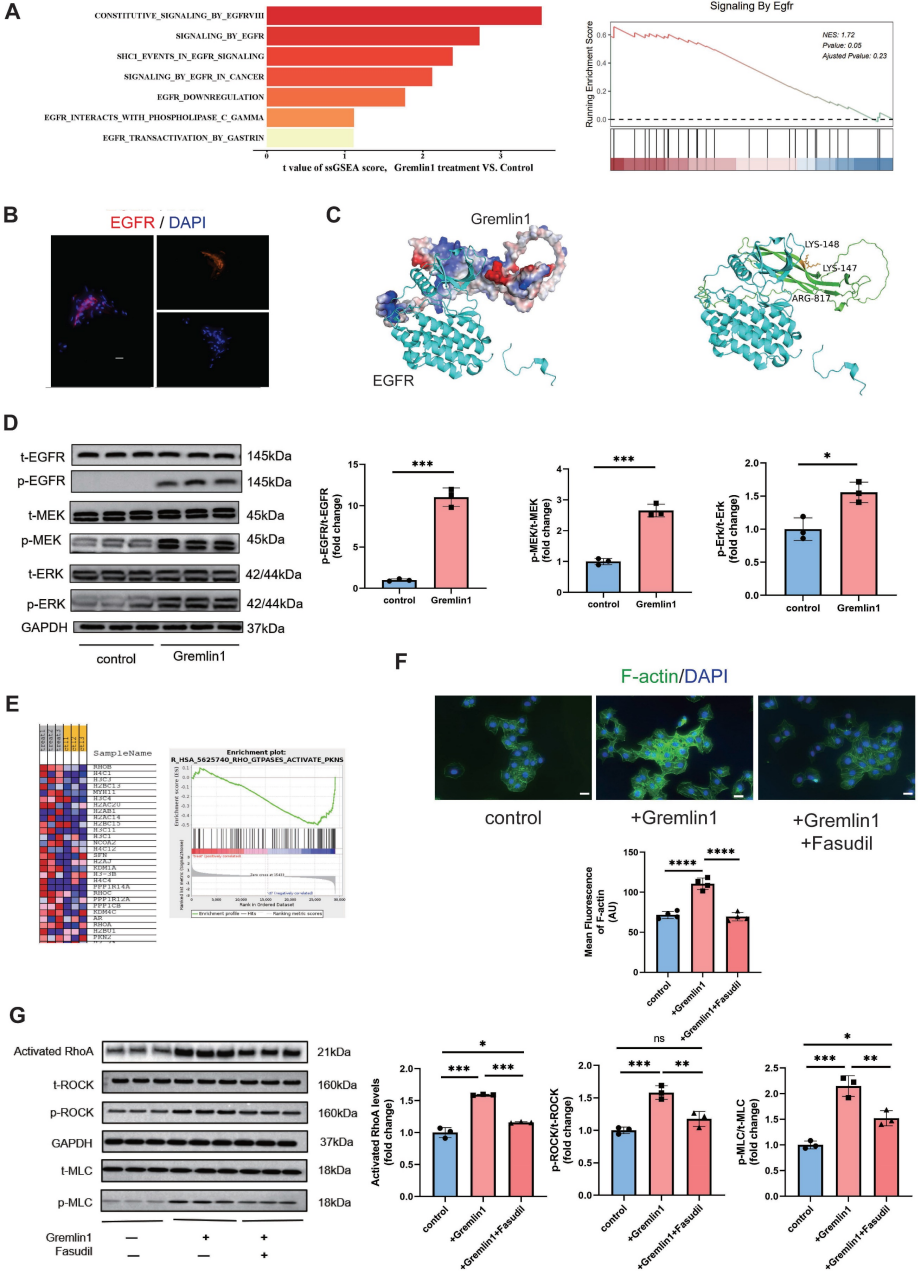
**Gremlin1/EGFR activated RhoA via EGFR/MEK/Erk signaling in HURECs. (A)** Gene set enrichment analysis (GSEA) of RNA-seq data demonstrating that the EGFR signaling was activated in under Gremlin1 stimulation. **(B)** Representative images of FVM stained for EGFR (red). Cell nuclei were counterstained with DAPI (blue). Two channels were merged. Scale bar, 20 μm. **(C)** Predicted interaction of protein structures of EGFR and Gremlin1 and key amino acid residues in the binding pocket between EGFR and Gremlin1. **(D)** Representative western blots and quantification of EGFR/MEK/Erk signaling. GAPDH was used as the internal control (n = 3 for each group). Student's* t* test was used to compare differences. **(E)** GSEA pathway analysis of control / Gremlin1 treated HURECs for Rho GTPase activation. Heat map displayed the expression of typical genes involved in RhoA/ROCK signaling pathway. **(F)** Immunofluorescence staining of F-actin (green) and DAPI (blue) in each treatment group. Scale bar, 10 μm. **(G)** Western blotting analysis of activated RhoA, p-ROCK, t-ROCK, p-MLC and t-MLC in HURECs treated with Gremlin1 or Gremlin1 + Fasudil for 48h. GAPDH was used as the internal control (n = 3 for each group). One-way ANOVA was used to compare the difference. *P<0.05, **P<0.01, ***P<0.001 compared with control group.

To explore the mechanism underlying Gremlin1 signaling, we carried out gene set enrichment analysis (GSEA) on bulk RNA-seq of HURECs after Gremlin1 treatment. GSEA revealed that EGFR signaling was significantly changed in the Gremlin1 treated group, consistent with published data showing that Gremlin1 acts as a ligand for EGFR (Figure 5A) 14. We also confirmed EGFR expression on fibrovascular membranes (FVMs) using immunofluorescence staining and we observed that EGFR was present in fibrovascular membranes (Figure 5B).

Interaction of protein structures and key amino acid residues in the binding pocket were performed to predict the combination of Grelmin1 and EGFR (Figure 5C). Gremlin1 has been reported to activate EGFR and its downstream molecules via phosphorylation 14. Therefore, we detected the expression of t-EGFR, p-EGFR, t-MEK, p-MEK, t-Erk and p-Erk by Western blotting analysis. As shown in Figure 5D and Figure 7A, Gremlin1 induced the phosphorylation of EGFR, MEK and Erk in HURECs and BV2 cells respectively.

EGFR-dependent activation RhoA/ROCK signaling pathway is reported to regulate cytoskeletal rearrangement 15, induce EndMT^16^, and promote organ fibrosis^17^. GSEA analysis revealed significant enrichment of Reactome terms related to Rho GTPases activity, in addition to a number of altered transcripts regarding to RhoA/ROCK signaling (Figure 5E). We found that 48h exposure to Gremlin1 up-regulated the activated RhoA, and the phosphorylation level of ROCK and MLC, the direct downstream proteins of RhoA while the expression of total ROCK and total MLC was unchanged (Figure 5G). Aside from MLC phosphorylation, ROCK can also mediate the polymerization of G-actin to F-actin. In addition, immunofluorescence staining revealed that markedly elevated F-actin (another downstream protein of RhoA/ROCK pathway) expression in HURECs treated with Gremlin1 (Figure 5F). The upregulation of phospho-ROCK and phospho-MLC were significantly attenuated when HURECs were cultured in Gremlin1 supplemented with Fasudil, a classic RhoA/ROCK inhibitor (Figure 5F, G). Collectively, these findings suggested that RhoA/ROCK signaling was activated in HURECs under Gremlin1 stimulation.

**Figure 6:**
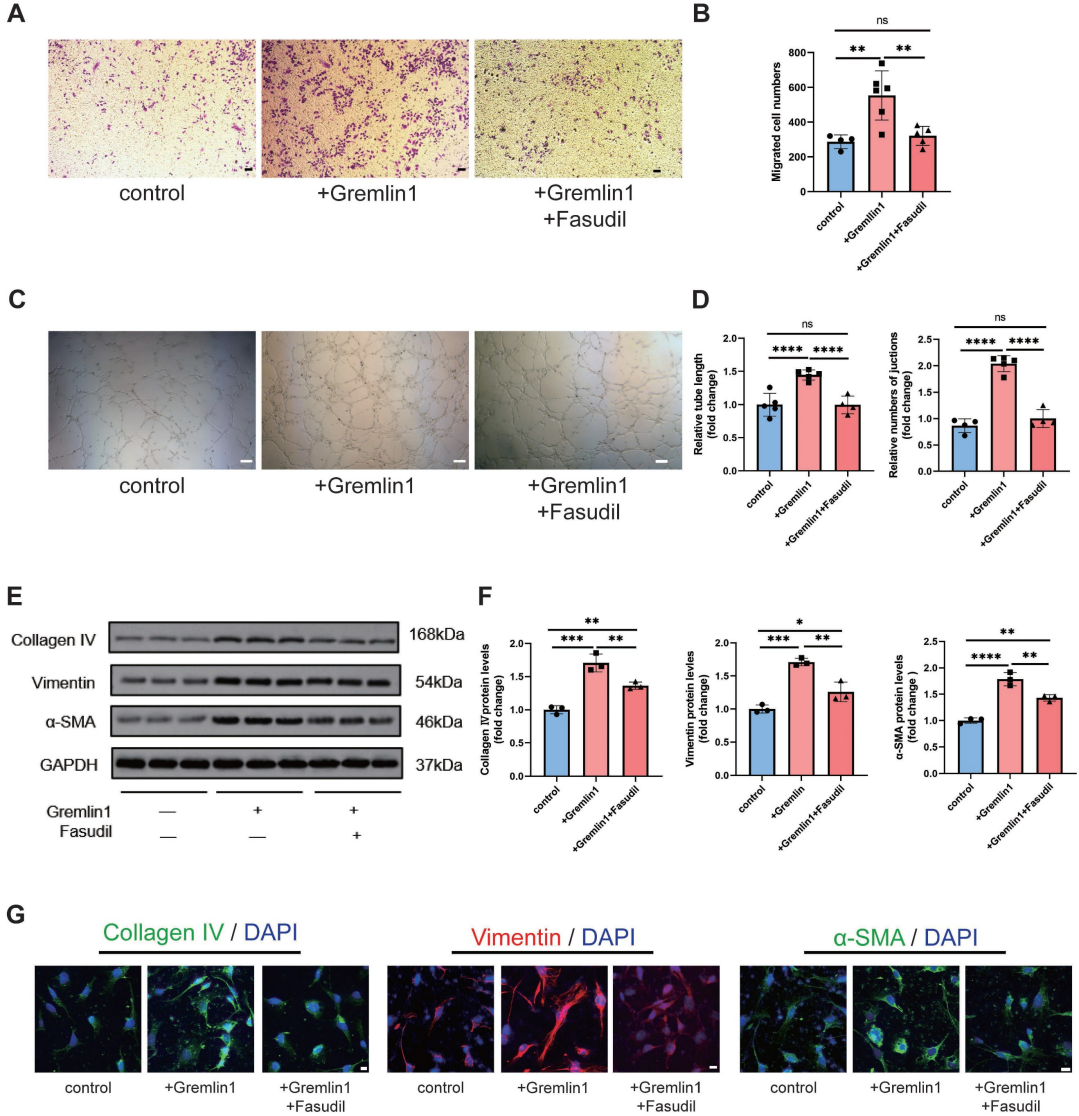
** The EndMT effect of Gremlin1 was dependent on activation of the RhoA/ROCK signaling pathway. (A-D)** Effects of Gremlin1 and Fasudil on the migration (A and B) and tube formation (C and D) in HURECs at 48h (n = 4-7 for each group). Scale bar, 50 μm. **(E, F)** Western blotting analysis of typical EndMT markers in HURECs treated with Gremlin1 or Gremlin1 + Fasudil for 48h. GAPDH was used as the internal control (n =3 for each group). **(G)** Representative images of immunostaining for Vimentin (red), Collagen IV (green), αSMA (green) and DAPI (blue) at 48h in ECM, ECM+Gremlin1 and ECM + Gremlin1 +Fasudil groups. Two channels were merged. Scale bar, 10 μm. One-way ANOVA was used to compare the difference. *P < 0.05, **P<0.01, ***P<0.001 compared with control group.

To further investigate the role of RhoA/ROCK pathway in Gremlin1-induced HURECs dysfunction, we also measured the migration, invasion, and tube formation of cells treated with Gremlin1 or Fasudil. The results revealed that Fasudil neutralized the proangiogenic effect of Gremlin1 (Figure 6A-D). Meanwhile, Western blotting analysis and immunofluorescence staining confirmed that the expression of mesenchymal markers Collagen IV, Vimentin and α-SMA in HURECs was upregulated by Gremlin1, but then reversed resecured by Fasudil (Figure 6E-G).

**Figure 7:**
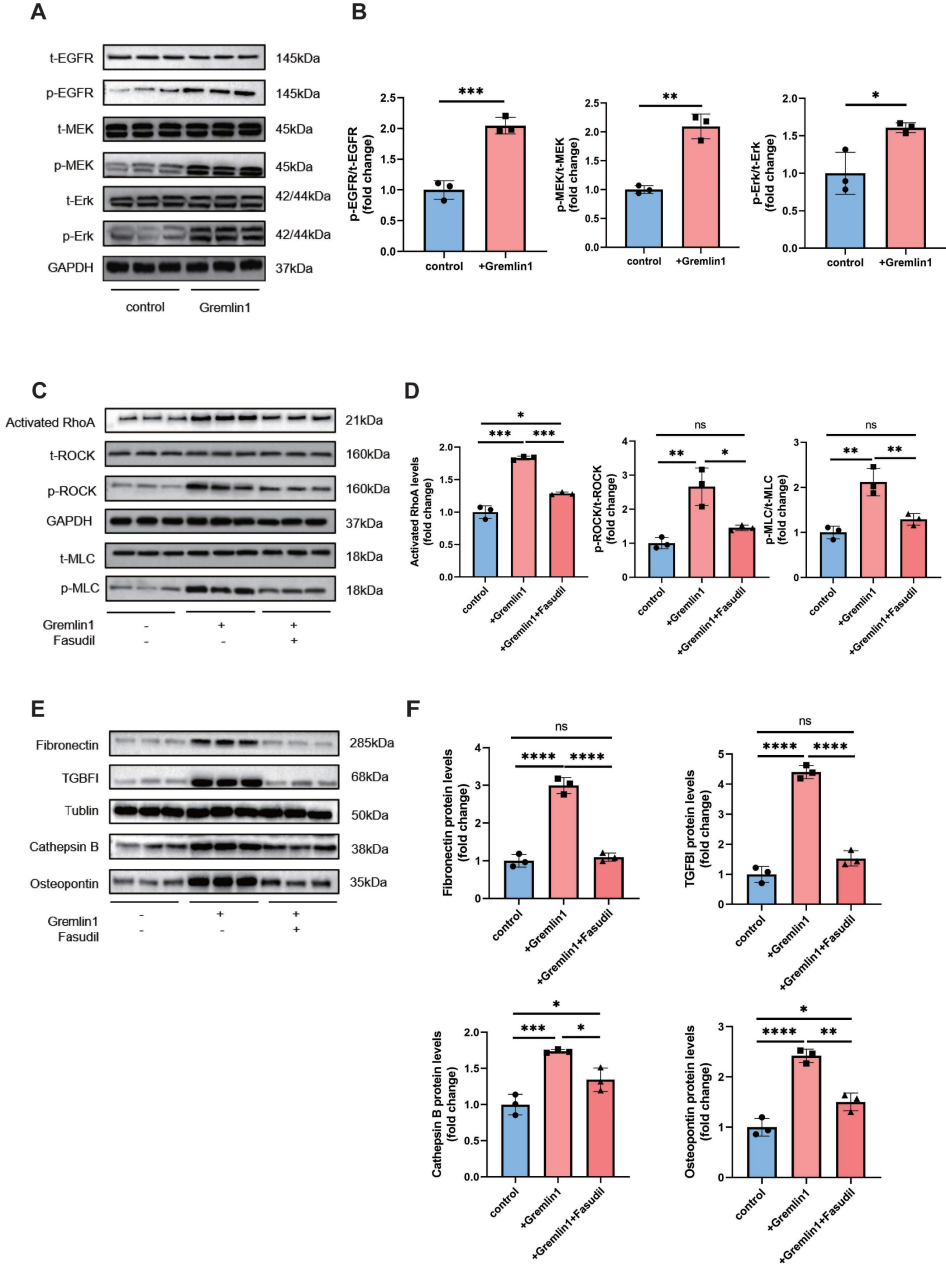
**Gremlin1/EGFR activated RhoA via EGFR/MEK/Erk signaling in BV2 cells. (A, B)** Representative western blots and quantification of Cathepsin B, Osteopontin, Fibronectin and TGFBI in BV2 cells treated with Gremlin1 or Gremlin1 + Fasudil for 48h. GAPDH was used as the internal control (n = 3 for each group). **(C, D)** Representative western blots and quantification of EGFR/MEK/Erk signaling. GAPDH was used as the internal control (n = 3 for each group). **(E, F)** Western blotting analysis of activated RhoA, p-ROCK1, t-ROCK, p-MLC and t-MLC in BV2 cells treated with Gremlin1 or Gremlin1 + Fasudil for 48h. Tublin was used as the internal control (n =3 for each group). One-way ANOVA was used to compare the difference. *P < 0.05, **P<0.01, ***P<0.001 compared with control group.

Similarly, we incubated BV2 microglia with Gremlin1 (200ng/mL) for 48h and found that EGFR/RhoA/ROCK pathway was also activated (Figure 7A-D). Western blotting analysis results verified the profibrotic effect of Gremlin1 was modulated by EGFR/RhoA/ROCK signaling, but was reversed by Fasudil (Figure 7E, F). Together, our findings demonstrated that the angiogenic effect of Gremlin1 in HURECs and profibrotic effect in microglia was mediated by the EGFR/MEK/Erk/RhoA/ROCK signaling pathway.

### Anti-Gremlin1 antibody inhibited retinal and choroidal angiogenesis and microglia-related fibrosis

**Figure 8:**
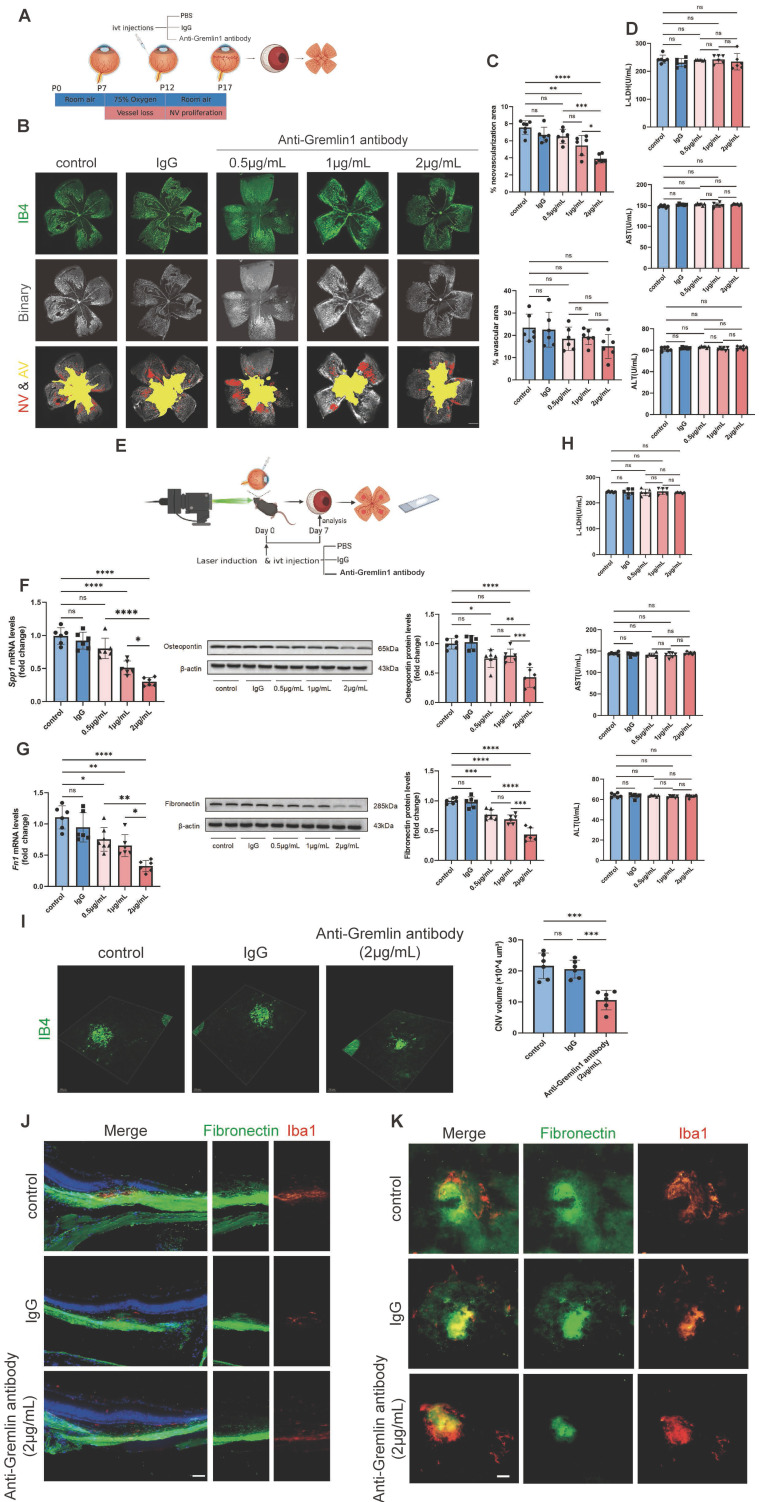
** Intravitreal injection of Gremlin1 antibody suppressed pathological angiogenesis. (A)** Experimental scheme. **(B)** Isolectin B4 staining was performed to examine pathological retinal neovascularization in whole-mount retinae at P17 in the OIR mice model. Scale bar, 500 μm. **(C)** Percentages of neovascularization and avascular areas were calculated and compared (n=6 for each group). **(D)** Toxicity analysis in OIR mice. **(E)** Schematic diagram of experimental procedures.** (F)** Analysis of *Spp1* mRNA (n=6, *Gapdh* was used as the internal control) and Osteopontin protein levels (n=6, β-actin was used as the internal control). **(G)** Analysis of *Fn1* mRNA (n=6, *Gapdh* was used as the internal control) and Fibronectin protein levels (n=6, β-actin was used as the internal control).** (H)** Toxicity analysis in CNV mice. **(I)** Isolectin B4 staining was performed to examine choroidal neovascularization in laser induced mouse CNV model. Choroidal tissue was scanned and reconstructed under a confocal microscope. **(J)** Quantification of the volume of CNV (n=6 for each group and 4 laser burns per sample for each group). **(K)** Wax-embedded sections were stained for Fibronectin (green), Iba1 (red) and DAPI (blue). Three channels were merged. Scale bar, 100 μm. (H) RPE/choroid flatmounts were stained for Fibronectin (green) and Iba1 (red). Two channels were merged. Scale bar, 50 μm. One-way ANOVA was used to compare the difference. **P<0.01, ***P<0.001 compared with control group.

To verify the potential therapeutic effect of Gremlin1 antibody, we examined the changes in retinal and choroidal neovascularization after intravitreal injection. Mice in the OIR model at P12 were intravitreally injected with PBS, IgG or Gremlin1 specific neutralizing antibody (Figure 8A). Compared with either PBS or IgG-treated groups, retinal angiogenesis was markedly attenuated after intravitreal injection of Gremlin1 antibody, with the most pronounced effect observed at the highest soluble concentration (2μg/mL) (Figure 8B, C).

Moreover, PBS, IgG or anti-Gremlin1 antibody was intravitreally injected into adult mice immediately after laser coagulation (Figure 8E). To test whether anti-Gremlin1 antibody exerts anti-fibrosis effects, we initially examined the expression levels of relevant key factors. The results demonstrated that as the antibody concentration increased, the expression of *Spp1*, Osteopontin (Figure 8F), *Fn1* and Fibronectin (Figure 8G) gradually decreased, indicating 2μg/mL as the optimal therapeutic concentration. We further confirmed the dual effects of Gremlin1 antibody at a concentration of 2μg/mL using immunofluorescence. After injection of anti-Gremlin1 antibody in CNV mice, IB4+ neovascularization volume shrunk by up to 21.35%, compared with that in vehicle controls or IgG group (Figure 8I). Cryosections from a CNV mouse eye were stained for Fibronectin(green) and Iba1 (red) and imaged. Gremlin1 antibody treatment decreased the Fibronectin deposit at the lesion site, compared to that of the control group (Figure 8J). Furthermore, subretinal fibrosis was further examined in RPE/choroid/sclera flatmounts using Fibronectin and Iba1 staining. Importantly, fewer area of fibrosis and microglia expressing fibronectin were observed in the subretinal space in the anti-Gremlin1 antibody-treated group (Figure 8K). Together, these findings indicated that anti-Gremlin1 antibody strongly inhibited ocular angiogenesis and microglia-related fibrosis *in vivo*. Furthermore, mice did not exhibit liver damage following antibody injection, indicating the safety of antibody usage (Figure 8D, H).

## Discussion

**Figure 9:**
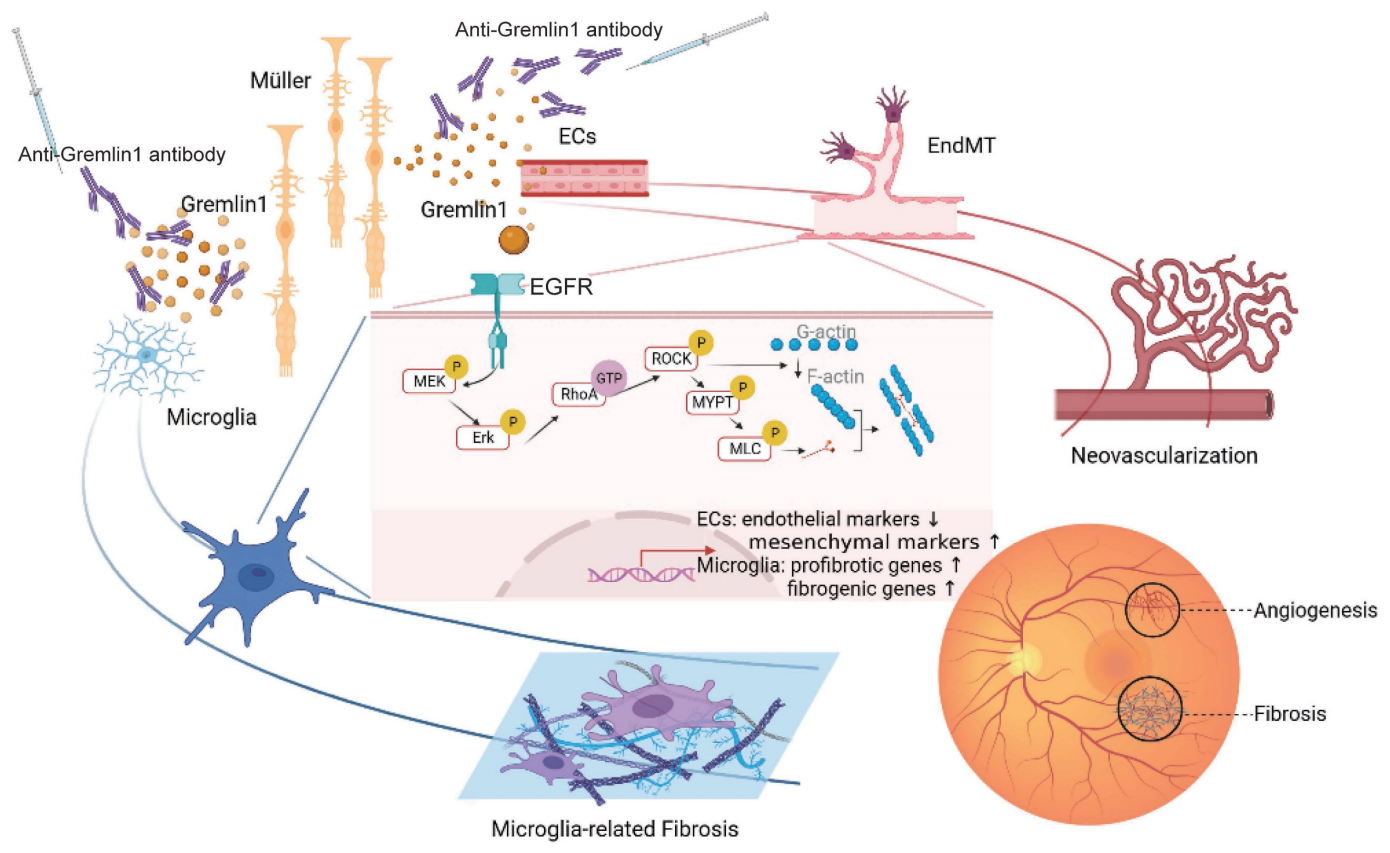
A schematic illustration depicting the molecular mechanism of Gremlin1 signaling in PDR.

In this current study, we reported that (a) the concentration of Gremlin1, which derives from Müller cells, was increased in vitreous fluid of PDR patients and in the retinae of ocular angiogenesis mouse models; (b) Gremlin1 promoted angiogenesis and microglia-related fibrosis via EGFR/MEK/Erk/RhoA/ROCK signaling axis; and (c) Gremlin1 antibody treatment provided a dual-effect of anti-angiogenesis and anti-fibrosis in OIR and CNV models. Collectively, our data revealed the function of Gremlin1 in ocular angiogenesis and the therapeutic potential of anti-Gremlin1 antibody in proliferative diabetic retinopathy (Figure 9).

We identified that Gremlin1 was harbored by Müller glial cells, indicating the critical role of Müller cells in maintaining retinal homeostasis. Previously, in addition to vasculopathy, recent studies have also shown retinal neuropathic changes in DR 18-20. As DR progresses, retinal ganglion cells (RGCs) undergo functional impairments 21, 22, axonal loss 23, 24 and eventually death 25, 26. Since RGCs cannot be replicated or regenerated, the damage of retinal neurons might lead to irreversible vision loss. The molecular mechanism of RGC apoptosis involves the cellular oxidative stress caused by high glucose and hypoxia environment^27^, ^28^, delayed clearance of advanced glycation end products (AGEs)^29^, ^30^, accumulation of glutamate^31^, ^32^, as well as proinflammatory factor release from abnormally activated Müller cells^33^, ^34^.According to previous studies, significant glial changes occur in the early progression of diabetic retinopathy in order to provide critical homeostatic and trophic support for both retinal neurons and vasculature. Babapoor-Farrokhran has demonstrated that Angiopoietin-like 4 is a novel angiogenic factor which expression was increased in hypoxic Müller cells 35. Yang proposed that endoplasmic reticulum stress of Müller cells is associated with vascular leakage and retinal inflammation 36. Recently, we also have unveiled the dynamic crosstalk between retinal Müller glial cells and endothelial cells 37. The current study further revealed the pathogenic role of dysfunctional Müller cells in DR. Therefore, more attention should be paid to the role of Müller in DR pathogenesis as well as its potential to become a therapeutic target.

Gremlin1 is a highly conserved secreted protein of 20.7 kDa and belongs to the BMP inhibitors family 6. By forming dimers with BMP2, BMP4 and BMP7, Gremlin1 prevents the binding of BMP to its receptor and inhibits the downstream phosphorylation of Smad1/5/8, thus regulating the BMP signaling pathway 38. Gremlin1 is not only essential for embryonic development, but has also been reported to be closely associated with diseases such as organ fibrosis, pathological angiogenesis and tumor progression. Although the relationship between Gremlin1 and diabetic retinopathy has not been deeply studied, its connections with angiogenesis and fibrosis have been reported. Zhang et al. reported that Gremlin1 promoted EndMT and angiogenesis in pulmonary arterial hypertension 39. Gremlin1 was also found to increase the secretion and remodeling of extracellular matrix in hepatic, pulmonary and diabetic nephropathy fibrosis. Zhang et al. found that renal fibrosis and inflammation of the diabetic nephropathy model mice were relieved after application of siGrem1^40^. Notably, the upregulation of Gremlin1 leads to insulin resistance and increased endoplasmic reticulum stress.^41^ It is obvious that insulin resistance is associated with diabetes, and endoplasmic reticulum stress in endothelial cells has been confirmed to be an inducer of angiogenesis.

Although previous understanding of Gremlin1 mainly focused on its role as a BMP inhibitor, Gremlin1 can also act through BMP-independent pathways. Park reported that Gremlin1 promotes proliferation of cancer cells through the EGFR pathway 14. Recently, a study published on *Nature* proposed that Gremlin1 can bind to FGFR on prostate cancer cells. By activating the MAPK pathway, Germlin1 drives tumor cells to acquire lineage plasticity and resistance to anti-androgen therapy 42. It has also been suggested that Gremlin1 may act as an inhibitor of macrophage migration inhibitory factor (MIF) in atherosclerosis 43. Huang et al. proposed in their study of diabetic nephropathy that a high-glucose stimulation could induce the secretion of Gremlin1 in mouse mesangial cells, which then activated the Erk pathway and promoted extracellular matrix deposition 44. These studies suggested the intricate connection between Gremlin1 and diabetic retinopathy, however, the underlying molecular mechanisms has not been fully explored.

Here, the dual effect of Gremlin1 is related to RhoA/ROCK signaling axis. Activated RhoA and its downstream proteins promoted the formation of actomyosin stress fibers, which is the structural basis for cell migration. On the other hand, activated ROCK could upregulate the expression of various fibrosis-related markers such as Collagen type IV and α-SMA, via MRTF/SRF signaling and YAP/TAZ/TEAD signaling 45-48. Mounting research suggests that sprouting endothelial cells may undergo partial and reversible EndMT during angiogenesis, since angiogenesis and EndMT share similarities, including cellular malformation and polarity, extension of filopodia, breakdown of cell-cell junctions, upregulated expression of mesenchymal markers, and extracellular matrix degradation. Through the RhoA/ROCK pathway, Gremlin1 can participate in the rearrangement of the cytoskeleton and the reconstruction of the extracellular matrix, promoting EndMT and angiogenesis. In fact, the regulation of EndMT by RhoA/ROCK has been reported. Activation of RhoA was reported to be related to the transformation of endothelial cells into cancer-associated fibroblasts through EndMT^49^. Further, oxidative stress can affect the late stage of EndMT via RhoA signaling pathway 50.

We have recently showed that microglia present on the PDR fibrovascular tissues and is understood to be essential in development of PDR. Furthermore, the activated microglia on FVMs presents both profibrotic and fibrogenic properties. The progression of PDR, to some extent, resembles the process of wound healing. During the proliferative phase of wound healing, M2-type macrophages produce a wide range of potent fibrogenic mediators and stimulate fibrosis response. Notably, RhoA/ROCK pathway has been demonstrated to regulate macrophage polarization. Xu et al. found that melatonin attenuated CNV by switching the macrophage/microglia polarization from M2 phenotype to M1 phenotype via inhibition of RhoA/ROCK signaling pathway 51. Zandi et al. discovered that a small molecule ROCK inhibitor reduced choroidal neovascularization 52. Consistent with previous researches, our present study confirmed that Gremlin1 promotes microglia to be profibrotic and fibrogenic via RhoA/ROCK axis.

In summary, our study provides a novel insight into the mechanisms by which Gremlin1 regulates both angiogenesis and fibrogenesis, encouraging further exploration of its therapeutic effects for ocular neovascularization diseases. Despite the scientific and rigorous findings we have obtained in the current work, there are still some limitations to be mentioned. Firstly, the clinical sample size was relatively small and more patients or other angiogenic retinal diseases will be more convincing if included. Besides, the most ideal control group should include symptomatic floaters patients to minimize the influence of retinal condition on the composition of vitreous humor. Finally, future studies are needed to verify these findings through Müller-specific interference in *Grem1* or employing conditional *Grem1* knockout mice.

## Material and Methods

### Patients

Vitreous humor from 9 PDR patients and 10 non-diabetic control patients was used for liquid suspension nano-chip analysis. For immunofluorescence staining, vitreous fibrovascular membranes (FVMs) were collected from patients previously diagnosed with DR.

The human studies were approved by the Ethics Committee of First Affiliated Hospital of Nanjing Medical University. Informed consent was acquired from each subject.

### Animal studies

All animal procedures were approved by the Institutional Animal Care and Use Committee at Nanjing Medical University and also in accordance with the ARVO Statement. This study employed rats, pups and adult female C57BL/6 mice that were housed in a normal experimental room in a 12 h light/dark cycle and with free access to water and food.

A model of oxygen-induced retinopathy (OIR) was established as described before 53, 54. At postnatal days 7 and 12 (P7-P12), the mouse pups were kept in a hypoxic chamber (75±2%) with their mother. In the following 5 days, pups and their mother were transferred to normoxic conditions. Intravitreal injections of PBS (0.138 mol/l NaCl, 0.0027 mol/l KCl, [pH 7.4]), Gremlin1 protein, IgG or Gremlin1 antibody were conducted in OIR mice at P12. The pups were anesthetized at P17 and their eyeballs or retinae harvested. The numbers of mouse pups in all groups are shown in the corresponding figure legends.

The laser-induced choroidal neovascularization (CNV) model was constructed as described before 55, 56. In brief, the mice were anesthetized and pupils dilated with 1% tropicamide. Two minutes after pupil dilation, four laser burns were made 2 disk diameters from the optical disk by 532 nm laser (Iris Radiation Systems, USA; power 100 mW, duration 100mS, spot size 75 μm). The formation of a cavitation bubble with no hemorrhage indicated the successful disruption of Bruch's membrane. The mice were subjected to intravitreal injections of PBS, Gremlin1 protein, IgG or Gremlin1 antibody immediately after photocoagulation. One-week after induction of laser burns, the mice were killed and their eyes were enucleated for further processing. Each treatment was performed in 6 mice, as shown in the figure legends.

### IVT injection

Intraocular injection was conducted in OIR pups (0.5μL) and CNV adult mice (1μL). In brief, the mice were anaesthetized with isofluorane, followed by pupil dilation with 1% tropicamide. Then, intravitreal injections were performed under a surgical microscope. A 33-gauge needle was inserted into the vitreous body from a 45° angle, and a 5μL Hamilton syringe was applied to deliver the solution. After injection, each eye was treated with an antibiotic ointment (Alcon, Tobrex ®Tobramycin 0.3%).

In accessing the role of Gremlin1 protein, rec-GREM1 (MCE, Cat#HY-P76378) was dissolved to 200 μg/mL as working solution. Gremlin1 antibody (Invitrogen Cat# PA5-47973, RRID: AB_2610125) and Goat IgG Isotype Control (Invitrogen Cat# 31245, RRID: AB_10959406) was purchased from Invitrogen and dissolved to 2μg/mL in PBS before intraocular injection.

### Cell culture

Human retinal endothelial cells (HURECs), purchased from Shanghai Institute of Biochemistry and Cell Biology, Chinese Academy of Science (Shanghai, China), were cultured in ECM medium (ScienCell, Cat#1001) added with 5% FBS, EC growth supplements (ECGS), 100 U/mL penicillin and 100 μg/mL streptomycin in a humidified atmosphere with 5% CO2 at 37 °C.

Primary Müller cells were isolated under the guidance of the method of Hicks and Cortois^57^. Briefly, the retinae were collected from P6 SD rats and stored in DMEM/F12 (Gbico, Cat#1320033) without FBS, then transferred to be digested in solution containing 0.1% pre-warmed Trypsin-EDTA (Gbico, Cat# 25200072) and 70U/mL collagenase1 (Solarbio Science & Technology Co., Cat#C8140), followed by an incubation at 37°C for 20 min. The digestion was terminated with DMEM/F12 complete medium supplemented with 10% FBS (Gbico, Cat#10100147C) and 1% penicillin-streptomycin (Gbico, Cat#15140148). Afterward, the cell suspension was moved to T25-cell culture flasks (Corning, Cat#3289) and cultured for 1 week, with the medium completely changed every 3 days. The cells were isolated and allowed to become confluent within 7-10 days and those in passages 2 were selected. Müller cells of the hypoxia group were incubated with 1% O_2_ for 48h.

The mouse microglia cell line BV2 (Pricella, Cat#CL-0493) were maintained in MEM supplemented with 10% FBS and 1% P/S at 37°C and 5% CO2 in a humidified incubator.

### Measurement of soluble Gremlin1 in vitreous humor

To detect the Gremlin1 concentration in patient vitreous humor, centrifugation was performed (10,000 rpm for 10 min at 4°C) and the supernatant was collected. Then, in accordance with the manufacturer's instructions, Vitreous Cytometric Bead Array (Bio-Rad) and Bio-Plex MAGPIX System (Bio-Rad) were applied to quantify Gremlin1 expression.

The concentration of soluble Gremlin1 was measured in cell culture medium after high glucose treatment using Enzyme-Linked Immunosorbent Assay (ELISA) kits (CUSABIO, Cat#CSB-E17688r). Prior to use, all materials and prepared reagents were equilibrated to room temperature. The cell culture medium was collected after high glucose stimulation, and centrifugated at 2000g for 10 min to remove debris. ELISA kits were used to measure Gremlin1 protein level in cell culture medium according to the manufacturers' protocols.

### Real-time reverse transcription polymerase chain reaction (RT-PCR)

Total RNA was extracted from mouse retina or different cell lines using Trizol reagent (Thermo Fisher Scientific, Waltham, MA, USA) and transcribed using *Evo M-MLV* RTase Enzyme Kit (Accurate Biology, Cat#AG11705). The primer sequences used for qPCR amplification are shown in Supplemental Table 1.

Quantitative real-time PCR was performed using SYBR qPCR premix (Accurate Biology, Cat#AG11718). Cycles included an initial denaturation at 95°C for 30 s, 40 cycles at 95°C for 5 s, 60°C for 30 s, and 72°C for10 min. The expression levels of all target genes were determined using the δδ Ct method and normalized with Gapdh expression on a per sample basis.

### Western blotting analysis

The total protein concentration of retinal or cellular lysates was determined by Bradford assay (Beyotime, Cat#P0012S) and 40μg protein was processed for the analysis. To detect activated RhoA, GTP-bound RhoA was collected from 2 × 10^7^ cells according to the manufacture's protocol (Cell Signaling, Cat#8820). Proteins were separated by sodium dodecyl sulfate-polyacrylamide gel electrophoresis, transferred onto nitrocellulose membranes, then blocked with 5% Bovine Serum Albumin (Beyotime, Cat#ST2249-5g) and probed at 4°C overnight with primary antibodies listed in Supplemental Table 2. HRBP-conjugated secondary antibodies (1:2000, Proteintech Cat#SA00001-1, Cat#SA00001-2) were used to detect primary antibodies. Each step was followed by three times of washing with TBST washing buffer for 10min. Western blots were visualized by enhanced chemoluminescence system (Clinx Science Instruments). ImageJ software (National Institutes of Health, Bethesda, MD, USA) was used to quantify the blots.

### Cell migration assay

Wound healing assay was performed to access endothelial cell migration as described previously 58. Confluent HUREC monolayers grown in 6-well plates were starved for 12 h in ECM - 0.5% FBS. Next, a scratch was created with a sterile 200 µl pipette tip. Having been washed with ECM, the culture medium was replaced with ECM added with 0.5% FBS containing different concentrations of Gremlin1 protein for additional 24 h. At indicated time points (0h and 24h), scratch closures were observed and photographed under a microscope with a CCD camera (DP70, Olympus). The area of the wounds was assessed using ImageJ software.

### Cell invasion assay

In the Transwell invasion assay, 100μL of Matrigel (Corning, Cat#356243) was used to precoat the upper Transwell chamber (Corning, polycarbonate membranes containing 8.0μm pore) at 37°C. The lower chamber was filled with 500 μL of complete ECM supplemented with 5% FBS. HURECs (1.0×10^5^) were suspended in 200 μL of ECM containing 0.5% FBS with or without 200 ng/ mL recombinant Gremlin1 protein (R&D Systems, Cat#5190-GR-050) or 100 μM Fasudil (MCE, Cat# HY-10341A). Then the cells were added to the upper chamber. After incubation at 37°C for 12 h, the upper chamber was fixed with 4% PFA and stained with crystal violet. The HURECs that had migrated to the chamber bottom were observed and counted under a light microscope equipped with a digital camera.

### Tube formation assay

In the tube formation assay, 150 μl of 4°C Matrigel (Corning, Cat#356243) was transferred to a pre-cooled 48-well culture plate. Allow the culture plate incubate at 37°C for half an hour. HURECs (1.0×10^5^) were suspended in 200 μL of ECM containing 0.5% FBS with or without recombinant Gremlin1 protein or Fasudil. After incubation at 37°C for 12h, the HURECs were harvested and fixed with 4% PFA. Endothelial tube length and numbers of junctions were observed under microscope.

### Cell proliferation assay

In EdU incorporation assay, 50 μL of 25 μM EdU medium was infused to HUREC culture in the 96-well plate. The cells were incubated for 2 h (Ribobio, Cat#C10310-1), then stained with Apollo 567 and Hoechst. Images were captured under a fluorescence microscope (Olympus BX51, Japan).

### Immunofluorescence

Vitreous fibrovascular membranes and eyeballs were fixed with 4% PFA and embedded in O.C.T(SAKURA, Cat#4583). 10μm thickness cryostat sections of retinal tissues were collected on superforst glass slides. For EGFR, Fibronectin and Iba1 visualization, the sections were washed with PBS and permeabilized with 0.3% Triton X-100 for 1 h. Then, the sections were incubated with blocking buffer (1% BSA plus 0.5% Triton X-100 in PBS) for 1 h at room temperature to block the unspecific binding of antibodies. Next, the sections were exposed to primary antibodies overnight at 4°C. Primary antibodies were listed in Supplemental Table 3. Having been washed with PBS, the corresponding secondary antibodies (1:200, Life Technologies Cat#A-11001; Cat#A-11002) were applied at room temperature for 2 h.

HURECs cultured on coverslips were fixed with 4% paraformaldehyde for 15 min and permeabilized with 0.3% TritonX-100 for 15 min. Then the cells were blocked in blocking buffer for 1 h at room temperature and incubated with primary antibodies overnight at 4°C. Primary antibodies were listed in Supplemental Table 3. Following washing with PBS was a 2-hour incubation with fluorescein-conjugated secondary antibodies.

To analyze retinal and choroidal angiogenesis, retinal or choroidal sheets were isolated from the eyes and fixed in 4% paraformaldehyde at room temperature for 30 min, incubated with FITC conjugated Isolectin B4 (1:100, Vector Laboratories Cat#B-1205) and flat-mounted. Tissues and slides containing cells were mounted with DAPI Fluoromount-G (SouthernBiotech, Cat#0100-20). Images were obtained with a Leica microscope.

### Fluorescence *in situ* hybridization

*Grem1* mRNA expression in retinal sections of control mice, OIR and CNV models was detected with fluorescent *in situ* hybridization kit (GenePharma). Eye tissues were fixed with FAS fixing solution at 4°C for 8h and sections of 4um were prepared.

After dewaxing and rehydration, 100uL of Proteinase K working solution preheated to 37°C was added to each section of paraffin slices. After sufficient washing and dehydration, sections were incubated with the FITC-labeled m*Grem* probe mixture for 12 hours at 37°C. After fluorescence* in situ* hybridization performance, the sections were then stained with anti-glutamine synthetase (1:100, Abcam Cat#ab64613, RRID: AB_1140869), ensued by incubation with fluorescein-conjugated secondary antibody (1:200, Abcam Cat#ab150116, RRID: AB_2650601) and DAPI Fluoromount-G (SouthernBiotech, Cat#0100-20). Fluorescence was imaged using a confocal microscope (Leica Stellaris STED, Germany).

### RNA extraction and high-throughput sequencing

Total RNA was isolated from samples using the Trizol (Invitrogen Corp, USA). Assessed with the ND-1000 Nanodrop (Thermo Scientific, USA), each RNA sample showed a purity with an A260:A280 ratio above 1.8 and an A260:A230 ratio above 2.0. Evaluated using the Agilent 2200 TapeStation (Agilent Technologies, USA), each sample had an integrity above 7.0. Ribosomal RNAs were separated from total RNA using EpicentreRibo-Zero rRNA Removal Kit (illumina, USA), and the purified RNAs were fragmented to approximately 200 bp. These fragments were then treated with first-strand and second-strand cDNA synthesis, followed by adaptor ligation and enrichment with a low-cycle according to the kit protocol of Illumina (NEB, USA). Having been confirmed using the Agilent 2200 TapeStation (Agilent Technologies, USA) and Qubit®2.0 (Life Technologies, USA), the ligated RNA library products were diluted to 10 pM for cluster generation in situ on the pair-end flow cell. Sequencing was performed on HiSeq3000 platform.

Raw RNA sequencing data were checked using the FastQC software. Adapters, invalid reads and low-quality reads were eliminated with Trimmomatic (VERSION 0.36). The clean reads were mapped with the mouse reference genome (VERSION mm10) using HISAT2 (VERSION 2.1.0). HTSeq (VERSION 2.1.0) was subsequently employed to convert aligned short reads into read counts for each gene model. Differentially expressed genes (DEGs) were assessed by DEseq2 with read counts as input, corrected by Benjamini-Hochberg multiple tests. DEGs were pinpointed according to fold change >2 and adjusted *P*-value <0.05. KOBAS (VERSION 3.0) was introduced to run Kyoto Encyclopedia of Genes and Genomes (KEGG) and GO enrichment analyses. A *P*-value <0.05 indicated statistical significance. For each sample, the expression data were then put in Gene Set Variation Analysis (GSVA).

### Statistical analysis

All data were expressed as the mean ± standard error of the mean. GraphPad Prism v9.0 software (GraphPad Software, Inc., La Jolla, CA, USA) was taken to carry out statistical analysis. A one-way ANOVA test followed by Bonferroni's comparison was used. Mann-Whitney test was used for between-group comparison. A* P*-value < 0.05 was considered statistically significant.

## Supplementary Material

Supplementary figure and tables.

## Abbreviations

VEGF: vascular endothelial growth factor
PDR: proliferative diabetic retinopathy
BMP: bone morphogenetic protein
EMT: epithelial-mesenchymal transition
EndMT: endothelial-mesenchymal transition
ECs: endothelial cells
FVMs: fibrovascular membranes
ERM: epiretinal membrane
OIR: oxygen-induced retinopathy
CNV: laser-induced choroidal neovascularization
FISH: fluorescence *in situ* hybridization
GS: glutamine synthetase
HURECs: human retinal endothelial cells
IB4: Isolectin B4
RGCs: retinal ganglion cells
AGEs: advanced glycation end products
MIF: migration inhibitory factor
