# Supplementary material for: Angiogenic and Fibrogenic Dual-effect of Gremlin1 on Proliferative Diabetic Retinopathy
Source: Int J Biol Sci. 2024 Jan 12;20(3):897–915. doi: 10.7150/ijbs.85735 (PMC10797694; doi:10.7150/ijbs.85735)
Supplement: Supplementary file 1 — Supplementary figure and tables. [file ijbsv20p0897s1.pdf]

Supplementary materials

Table S1. The primer sequences for mRNA analysis.

Target mRNA	Forward primer	Reverse primer
Mouse *Gapdh*	5'-CAGTGGCAAAGTGGAGATTGTTG-3'	5'-TCGCTCCTGGAAGATGGTGAT-3'
Mouse *Grem1*	5’-GGGACCCTACTGCCAACAG-3’	5’-TTTGCACCAATCTCGCTTCAG-3’
Rattus norvegicus *Gapdh*	5’-GAAGGTCGGTGTGAACGGAT-3’	5’-CCCATTTGATGTTAGCGGGAT-3’
Rattus norvegicus *Grem1*	5’-GCGCAAATACCTGAAGCGAG-3’	5’-CTGTTTCCGCTGGTGTTTGG-3’
Human *GAPDH*	5’-GGAGTCCACTGGCGTCTTCA-3’	5’-GTCATGAGTCCTTCCACGATACC-3’
Human *CDH5*	5’-CTTCACCCAGACCAAGTACACA-3’	5’-TGTTGGCCGTGTTATCGTGA-3’
Human *PECAM1*	5’-AACAGTGTTGACATGAAGAGCC-3’	5’-TGTAAAACAGCACGTCATCCTT-3’
Human *VIM*	5’-GACGCCATCAACACCGAGTT-3’	5’-CTTTGTCGTTGGTTAGCTGGT-3’
Human *COL4A1*	5’-GGACTACCTGGAACAAAAGGG-3’	5’-GCCAAGTATCTCACCTGGATCA-3’
Human *ACTA1*	5’-GGCATTCACGAGACCACCTAC-3’	5’-CGACATGACGTTGTTGGCATAC-3’
Mouse *Spp1*	5’-ATCTCACCATTCGGATGAGTCT-3’	5’-TGTAGGGACGATTGGAGTGAAA-3’
Mouse *Lgals3*	5’- GGAGAGGGAATGATGTTGCCT-3’	5’- TCCTGCTTCGTGTTACACACA-3’
Mouse *Ctsb*	5’- CAGGCTGGACGCAACTTCTAC-3’	5’- TCACCGAACGCAACCCTTC-3’
Mouse *Fn1*	5’- ATGTGGACCCCTCCTGATAGT-3’	5’-GCCCAGTGATTTCAGCAAAGG-3’
Mouse *Tgfbi*	5’- CGCCAAGTCACCCTACCAG-3’	5’- TGCACAGCACATACATTGGGG-3’

Mouse *Spar*	5'- GTCGCAGACCGAAAGACCTG-3'	5'- CGACGCATGTAGAAGTCATCG-3'

Table S2. The antibodies used for Western blotting.

Antigen	Catalog#	Manufacturer	Dilution
Gremlin1	sc-515877	Santa Cruz Biotechnology, Dallas, USA	1:500
β-actin	BL005B	Biosharp, Shanghai, China	1:5000
Vimentin	ab92547	Abcam, Cambridge, UK	1:1000
Collagen type IV	55131-1-AP	Proteintech, Wuhan, China	1:1000
Smooth muscle actin	sc-53142	Santa Cruz Biotechnology, Dallas, USA	1:1000
Tublin	66031-1-Ig	Proteintech, Wuhan, China	1:1000
EGF Receptor	4267	Cell Signaling, Boston, USA	1:1000
Phospho-EGF Receptor	3777	Cell Signaling, Boston, USA	1:1000
MEK1/2	9122	Cell Signaling, Boston, USA	1:1000
Phospho-MEK1/2	9154	Cell Signaling, Boston, USA	1:1000
p44/42 MAPK	9102	Cell Signaling, Boston, USA	1:1000
Phospho-p44/42 MAPK	4370	Cell Signaling, Boston, USA	1:1000
Rock-2	sc-100425	Santa Cruz Biotechnology, Dallas, USA	1:500
ROCK2 (phospho Ser1366)	GTX122651	GeneTex Biotechnology, State of Texas, USA	1:500
Myosin Light Chain 2	ab79935	Abcam, Cambridge, UK	1:500
MLC (phospho S20)	ab2480	Abcam, Cambridge, UK	1:500
Fibronectin	ab268020	Abcam, Cambridge, UK	1:1000
TGFBI	ab170874	Abcam, Cambridge, UK	1:1000
Cathepsin B	ab214428	Abcam, Cambridge, UK	1:1000
Osteopontin	ab63856	Abcam, Cambridge, UK	1:1000

Table S3. The primary antibodies used for immunofluorescence.

Antigen	Catalog#	Manufacturer	Dilution
EGFR	ab52894	Abcam, Cambridge, UK	1:100
Fibronectin	ab268020	Abcam, Cambridge, UK	1:100
Iba1	ab178846	Abcam, Cambridge, UK	1:100
Vimentin	ab92547	Abcam, Cambridge, UK	1:100
Collagen type IV	sc-59814	Santa Cruz Biotechnology, Dallas, USA	1:100
Smooth muscle actin	sc-53142	Santa Cruz Biotechnology, Dallas, USA	1:100

Figure S1.

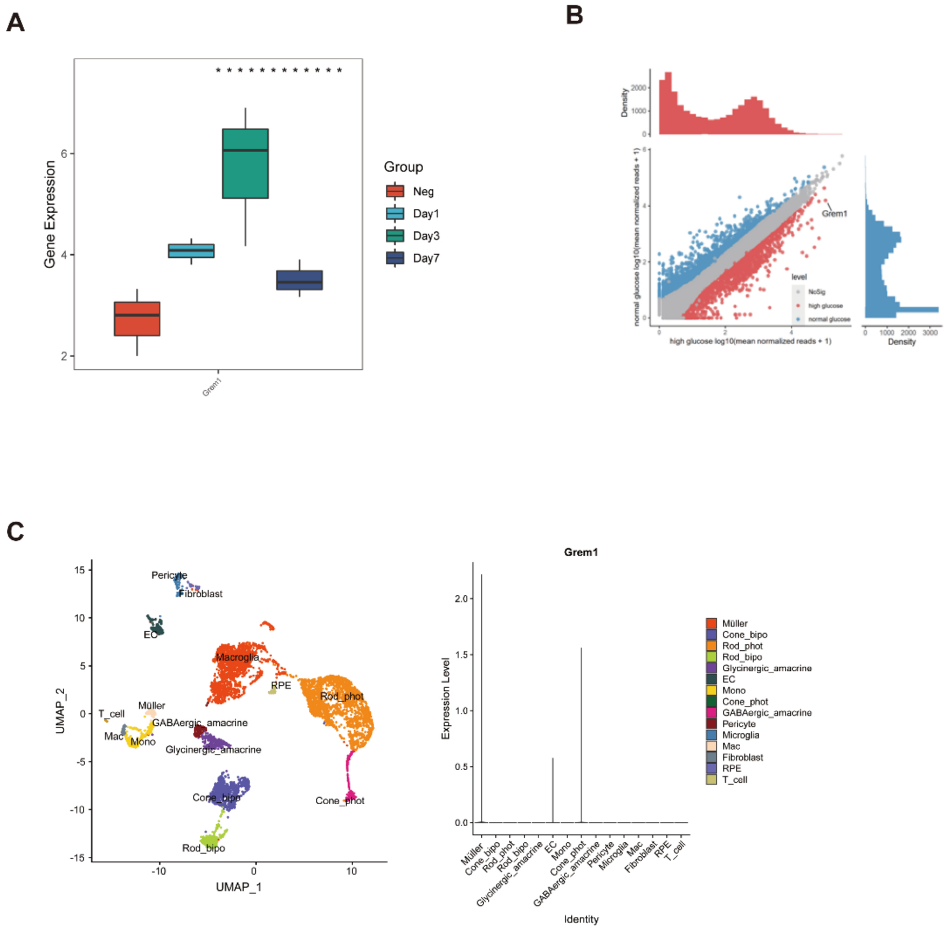

Figure S1. **Bioinformation analysis of *Grem1* expression. (A)** *Grem1* expression levels in Müller cells after laser coagulation. **(B)** Scatter plot using the log2 fold change to visualize DGEs between Müller cells treated with normal glucose (5.5 mM) and high glucose (25 mM) for 48h. The *Grem1* gene was labeled. Definition of DEGs: abs (log2 FC) > 1.5 & adj. p-value < 0.05. **(C)** Fifteen cell types were detected from single-cell sequencing analysis of Akimba mice retina. *Grem1* was mainly expressed in retinal Müller cells of Akimba mice.
